# A tribute to the memory of professor Alexander K. Popov

**DOI:** 10.1515/nanoph-2022-0655

**Published:** 2022-11-14

**Authors:** Gennady Tartakovsky, Alexei V. Sokolov, Mikhail Ivanov, Vasily G. Arkipkin, Sergey A. Myslivets, Boris Luk’yanchuk, Alexandra Boltasseva, Vladimir M. Shalaev

**Affiliations:** Advanced Systems &Technologies, Inc., Irvine, CA, USA; Institute for Quantum Science and Engineering, Department of Physics and Astronomy, Texas A&M University, TX 77843, USA; Max Born Institute, 12489 Berlin, Germany; Department of Physics, Humboldt University, 12489 Berlin, Germany; Blackett Laboratory, Imperial College London, SW7 2AZ London, UK; Kirensky Institute of Physics, Federal Research Center KSC SB RAS Krasnoyarsk, Russia; and Institute of Engineering Physics & Radio Electronics, Siberian Federal University, Krasnoyarsk 660041, Russia; Nanophotonics Department, Faculty of Physics, M.V. Lomonosov Moscow State University, Leninskie Gory 1, bldg 2, 119991 Moscow, Russia; School of Electrical and Computer Engineering, Birck Nanotechnology Center, Purdue University, West Lafayette, IN, USA

**Figure:**
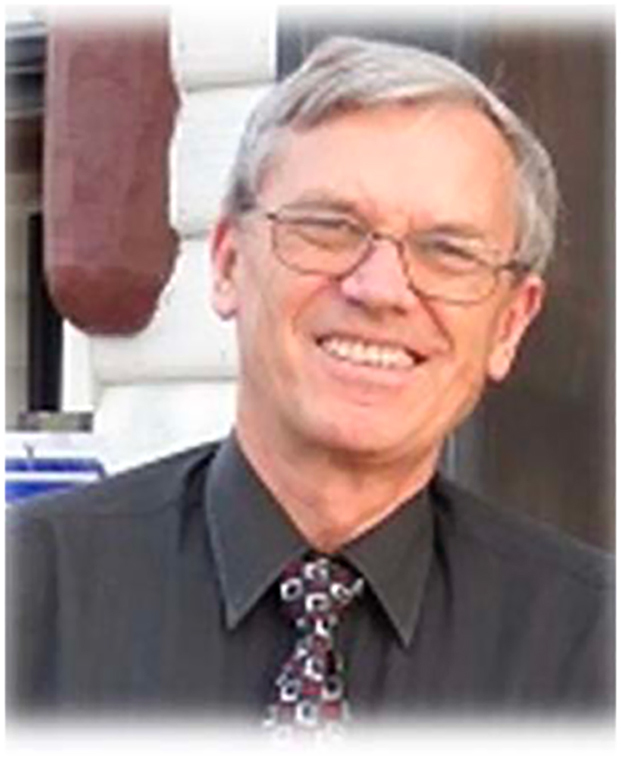


## 1 Introduction

Alexander (Alex) K. Popov, OPTICA (former Optical Society of America) Fellow and a former research professor at Purdue University passed away on March 31, 2022, at the age of 80, after years of struggle with a serious illness. Professor Alex Popov was a leading figure in quantum optics, nonlinear spectroscopy, and nonlinear optics, and made truly pioneering contributions to those fields. This includes quantum interference of atomic states in strong electromagnetic fields, high-order harmonic generation for IR, UV, and VUV light in gases and atomic vapors, optics of colloidal aggregates, theory of nonlinear optical phenomena in negative-index metamaterials as well as several other important contributions, as detailed in the sections below.

Professor Popov’s professional career spanned nearly 60 years. He studied physics at the Tomsk State University (Russia), where he obtained a Master of Science degree in theoretical optics in 1963. His MSc work was performed under the guidance of a famous Soviet physicist A. P. Kazantsev who made seminal contributions to the field of laser interaction with atoms and specifically to a theory of radiation pressure and laser cooling. In 1967, Alex Popov obtained his PhD degree in Physics and Mathematics from Krasnoyarsk Institute of Physics of the Russian Academy of Sciences. Notably, he completed his PhD project on “Nonlinear Theory of the Gas Laser” to a large extent on his own, without a formal thesis advisor. Well ahead of his time, in this and later works Alex Popov reported seminal discoveries of various important phenomena in so-called Doppler-broadened media interacting with strong electromagnetic fields. Later, Dr. Popov conducted his postdoctoral research at the Novosibirsk Institute of Semiconductors, Novosibirsk Research Center of the Russian Academy of Sciences (1967–1970) under the guidance of another famous Russian scientist Sergey G. Rautian, one of the pioneers of nonlinear optical spectroscopy. Popov and Rautian’s collaborative work resulted in critical contributions to the field and served to a high degree as a precursor for later developed important directions of lasing without inversion and electromagnetically induced transparency.

In 1976 Alex Popov founded the Laboratory of Coherent Optics in the Kirensky Institute of Physics of the Siberian Branch of the Russian Academy of Science. Many researchers, including some of the authors of this perspective (VGA, SAM and VMS) have been trained and conducted their studies in this world-class laboratory well known throughout the world for many pioneering studies in various areas.

Prof. Alex Popov has authored more than 500 articles during his research career and wrote several books, including “Laser-Induced Resonances in Continuum” (1981), with Yu. I. Heller, and “The Introduction to Nonlinear Spectroscopy” (1983, Novosibirsk, Nauka), which was based on his course of lectures taught in the Krasnoyarsk State University. He mentored numerous Ph. D students from 1970 until he left Russia in 2000 and joined the University of Wisconsin at Stevens Point, as a research professor of physics. Since 2008 he had continued his work as a research professor at Purdue University until his retirement in 2018.

Many of Professor Popov’s students and collaborators became well-known scientists working in various leading research centers in the world. Alex Popov was a great teacher. He liked to repeat to his students and younger researchers his favorite proverb “Not God but Man Makes Pot and Pan” and always encouraged them not to be scared and attack the most exciting and challenging problems. He was also a visionary scientist. For example, his early seminal studies on nonlinear interference effects in atoms interacting with strong electromagnetic fields were well ahead of the time and have later been developed into several active research areas, including lasing without inversion, light-induced continuum structures, electromagnetically induced transparency, efficient generation of extreme UV light, and stabilization of atomic states. These areas still keep attracting the best researchers today.

Below we outline some of the research areas, where Professor Alexander K. Popov made seminal contributions; all these fields remain very active, with many new discoveries to come. We also include a concluding section describing Alex Popov as a fine and unique person, with broad and diverse interests in many areas ranging from physics, to philosophy, to music, and literature.

This Introduction Section is written by Gene Tartakovsky and Vlad Shalaev. Section 2 below is written by Alexei Sokolov; 3 – by Misha Ivanov; 4 – by Gene Tartakovsky; 5, 6 and 7 – by Vlad Shalaev; and Section 8 – by Boris Luk’yanchuk (translated and edited by Misha Ivanov). Alexandra Boltasseva edited the whole paper; Vasily Arkhipkin and Sergey Myslivets provided useful comments and references.

## 2 Nonlinear interference effects in atoms interacting with strong EM fields

**Figure 1:**
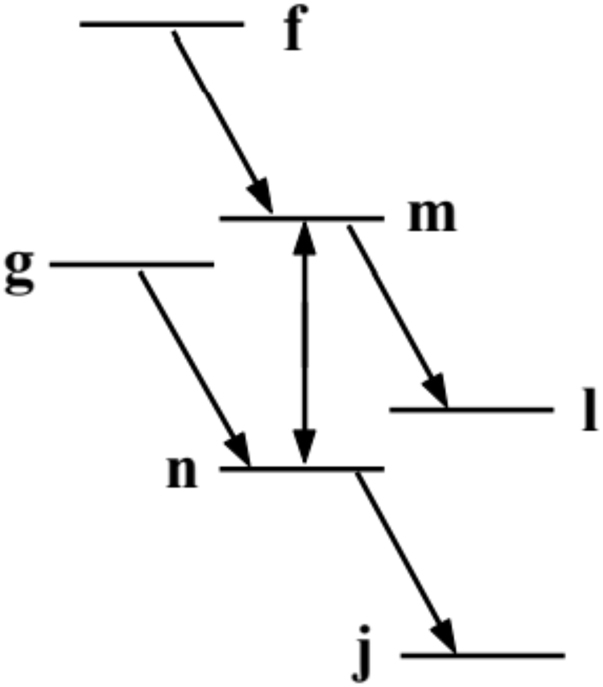
Level diagram for two monochromatic fields interacting with an atom. One strong field is resonant with the *m* − *n* transition; the other field probes the adjacent transition. Diagram reproduced from [1].

Early in his research career, Alex Popov produced prescient theoretical work on nonlinear interference effects in atomic emission and absorption. This work started during his ‘postdoc’ years at the Novosibirsk Institute of Semiconductors, with Sergey G. Rautian, one of the pioneers of nonlinear optical spectroscopy. Dr. Popov and his colleagues (T. Ya. Popova, S. G. Rautian, and R. I. Sokolovskii) considered what would happen if a sufficiently strong coherent laser beam drives an atomic transition. When an ensample of such atoms is ‘probed’ at a frequency that corresponds to a transition adjacent to the driven one (see Figure 1), remarkable things were predicted to occur. As you can see from the figure, the authors were aiming at describing the situation in a most generalized way. Every possible level configuration (Λ, V, ladder) was included in the analysis.

The authors analyzed the scheme where “the probability of absorption or emission of photons turns out to depend not only on the level populations but also on the polarization induced by the external field, i.e., on the nonlinear interference effect (NIEF). It is this effect that is responsible for causing the spectral densities of Einstein coefficients of absorption or emission to be different frequency functions, leading to characteristic changes in the pure emission or absorption” [1]. Their detailed analysis supported, for example, a prediction of light amplification without population inversion. Clearly, this work was many years ahead of its time. Fourteen years later, Alex Popov’s colleagues Arkhipkin and Heller demonstrated theoretically that an atomic system that exhibits (destructive) Fano interference in absorption does not show such interference in emission [2], leading to a possibility of inversionless gain. Their brief (2.5-page long) paper included only nine references, and even though it referred to some of Popov’s work and referred to a book referencing Popov’s work, it failed to refer to the 1969 paper [1]. Five more years later, Harris working on a related problem, analyzed interference of two lifetime-broadened autoionizing states [3], and discussed the application of his findings to lasing without inversion. He gave credit to Arkhipkin and Heller 1983 paper and stated the connection between his striking results and their work. However, at the time of his 1988 submission, Harris was unaware of Popov’s 1969 work (he learned of it years later). In 1989, Scully and his colleagues [4, 5] realized that the quantum interference scheme can be implemented without recourse to autoionizing states; they followed a route that was conceptually connected to the work of Popov and Rautian, showing how one can use atomic coherence between a pair of states to get lasing without inversion. Scully et al. relied on quenching of absorption via coherence generated in a ground state doublet (as in the case of population trapping [6, 7]) via an external radio-frequency field [4, 5]. Independently from the US counterparts and concurrently with Harris, in 1988 Kocharovskaya and her colleagues [8, 9] produced their theoretical prediction of lasing without inversion based on atomic coherence, coherent population trapping and quantum interference. What followed was a period of tremendous activity and interest in inversionless lasing. In spite of all the interest and activity in this field, the original 1969 paper by Popov and team remained overlooked for a number of additional years.

The ensuing decades saw a remarkably broad range of developments, based on the basic physics of atomic (and later molecular) coherence and quantum interference. Observation of electromagnetically induced transparency, which renders a medium transparent within a narrow spectral range around an absorption line, led to new opportunities for enhanced nonlinear optical conversion [10, 11]. The narrow-band transparency of an otherwise strongly absorbing medium is inevitably accompanied by extreme dispersion and therefore leads to “slow light” [12, 13]. By applying time-varying control-laser fields, researchers were able to “stop” or “freeze” light altogether, as well as to produce a reversible transfer of the quantum state between light and metastable collective states of matter, with applications to quantum memory [14, 15]. Molecular coherence was found to result in broadband Raman generation and enabled compression of single-cycle optical pulses through what was termed molecular modulation [16, 17]. That same quantum coherence in a molecular ensemble led to a laser spectroscopic technique for rapid identification of biological species [18], in dilute samples and down to the nanoscale, to the single-molecule limit [19, 20]. Last but not least, in a counterintuitive application of quantum coherence and interference phenomena to thermodynamics, it was shown that one can extract work from a single heat bath via vanishing quantum coherence [21].

## 3 Laser-induced-resonances in continuum

### 3.1 The music of the physics of Alexander Popov

*How long it takes to go*

*Down tricky winding roads,*

*Through the cliffs and canyons of the foggy Sierra …*

*All is silent in this world,*

*Just the hoofs of trusty donkey*

*Toss the pebbles off the path that’s oh-so-narrow …*

From “The Spanish Song” by Novella Matveeva

Writing about Alexander Kuz’mich Popov – AK for short – in the past tense is unnatural. His soft smile and his gentle manner are as alive and as striking today as they have been for decades, and they will always stay with us … but his work is done, and now it is time to take stock.

It is customary to say that so- and so-has made many important (or significant, or fundamental, whatever fits best) contributions to science. This is certainly true for AK and his physics, but so much more hides behind the list of his discoveries.

What matters is not only that AK has pioneered many key ideas and concepts in light–matter interaction, such as lasing without inversion (e.g. [1, 22], [23], [24], [25], [26], [27], [28], [29], [30], [31]), light-induced continuum structures (e.g. [2, 25, 26, 28, 32, 33]), stabilization of excited states against photo-ionization in intense light fields and electromagnetically induced transparency (e.g. [24, 25, 30, 31]), the nonlinear optical response of fractals and metamaterials (e.g. [34], [35], [36], [37]), and more, but also ***how*** he has done it – with artistry, with a blend of mathematical rigor and elegant simplicity.

His physics is like a symphony, where a theme is played out again and again, as it is being developed from its most basic form towards increasing complexity, and then, when the original melody seems to be lost in the complex dialogue of many (mathematical) instruments, it suddenly reappears again in its simple beauty. And as it often happens with a beautiful melody, his physics emerges again and again in new contexts, even those AK never studied. Such is the power of fundamental ideas.

The first time I heard the name of AK Popov was in the mid-eighties of the last century, in deep winter, in a snow-covered Voronezh, at the all-Soviet conference on multiphoton processes. I was still an undergraduate student then, and there I learned one of the most remarkable ideas in quantum optics: the idea that one can break the seemingly immovable balance between stimulated emission and absorption, appearing to violate the fundamental thermodynamics laws. This surprising effect is rooted in quantum interference, and it has taught us that one can amplify light propagating through a medium without populating the upper, emitting state, more than the lower, absorbing state, achieving lasing without population inversion.

**Figure 2:**
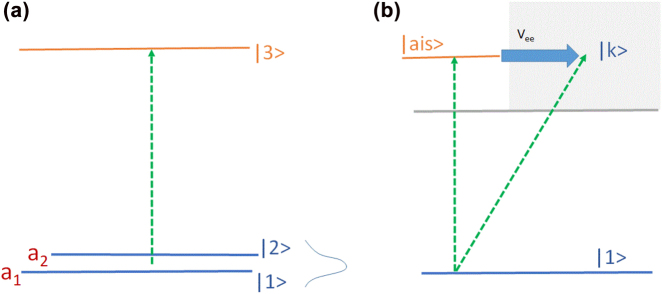
Two basic schemes breaking the balance between stimulated emission and absorption, leading to lasing without population inversion. Panel (a): Coherent superposition of two lower states can lead to destructive interference in absorption when the relative phase between the transitions from them is 180°. Panel (b): Interference of the two pathways to the continuum state |k⟩, directly along the diagonal and indirectly via |ais⟩ can also be destructive, suppressing absorption from the state |1⟩ but not the emission from the |ais⟩ to |1⟩.

The talent of AK Popov was to distill this striking idea into two most basic forms, two most elegant examples: a three-level system (e.g. [1, 22, 23], see also [38]) and a Fano resonance (Figure 2) (e.g. [2, 24, 25, 32]). In Voronezh, I have learned the story with the Fano resonance directly from Yuri Heller as it was told in [2], but let me start with the three-level system in Figure 2(a), because 40 years later its core idea and its realization in molecules [29] became central to my own work [39] on a totally unexpected phenomenon: lasing during laser filamentation in the open air.

If a short pulse incident onto a medium has enough spectral bandwidth to cover absorption from both states |1⟩ and |2⟩ to the same upper state |3⟩, a seemingly innocent thing would inevitably happen, no matter whether you like it or not: If both frequencies *ω*_13_ and *ω*_23_ are present in your incident pulse, both transitions will be induced simultaneously. Then, if a coherent superposition of the states |1⟩ and |2⟩ is prepared, the two absorption pathways will inevitably interfere. And then, if the superposition is $\left[\left|1\right\rangle -\left|2\right\rangle \right]/\sqrt{2}$ , the interference will be destructive, leading to the suppression of both transitions. Emission, however, does not suffer the same fate – it can proceed to the orthogonal superposition $\left[\left|1\right\rangle +\left|2\right\rangle \right]/\sqrt{2}$ . Thus, the interference of the two pathways may break the balance between absorption and emission, leading to gain even in an inversionless medium.

Forty years after learning this story, I have discovered that it finds its continuation when an intense laser pulse propagates in the air. Such intense light spins air molecules, particularly nitrogen, dominant in the air. In quantum lingo, a coherent superposition of rotational states is inevitably created, opening the chance for the scheme in Figure 2(a) to show up when the slowly evolving quantum phase between the adjacent rotational states approaches 180°. In this temporal window, radiation generated during emission from an excited electronic states of a spinning nitrogen molecule (in fact, molecular ion) will be amplified without ever realizing electronic population inversion relative to the ground electronic state of the same molecular ion.

The Fano resonance in Figure 2(b) presents another fundamental example – the second opening theme in the AK symphony. It is also ruled by quantum interference. Here, absorption of a photon in Figure 2(b) brings the electron to the continuum via two possible routes: directly along the diagonal or indirectly, making the right turn when going through the bound state labelled |ais⟩. How the two paths interfere, constructively or destructively, depends on the atomic structure given to us by nature and on the exact energy within the famous Fano line-shape. Yet, as long as there is energy where the destructive interference occurs, absorption dies there and then, opening the door to inversionless amplification of light.

How many of us had a chance to discover an idea that seemed to violate basic laws? And if you, my reader, did have such luck, what would you do next? Here, again, AK has taught us a lesson: not only did he not stop; he decided to confront another wall. I have just described this wall, and in case it went unnoticed, let me repeat it again: “How the two paths interfere, constructively or destructively, *depends on the atomic structure given to us by nature and on the exact energy within the famous Fano line-shape.*” Thus, one appears unable to choose what photon energy to amplify: the energies of autoionizing states are prescribed by nature, together with all the relevant transition amplitudes, their phases, and the position of the Fano minimum. How incredibly disappointing! Or is this wall yet another mirage, just like the impossibility of inversionless amplification appeared to be, only to be taken down from its “thermodynamic pedestal?”

Light can be used to move electrons and distort electronic clouds, shifting the quantum states given to us by Nature. With light, their energies can be raised by an integer number of photons, forming the so-called Floquet ladder of a “dressed atom”. Then, if we are missing a bound state embedded into continuum at the desired energy, why not use light to create it there? Why not add a photon to some excited state below the ionization threshold, raising it into continuum? This is how light-induced continuum structures have been invented [25, 32, 33, 40], with the position of the light-created autoionizing-like state tunable by changing the frequency of the “dressing” light and selecting the desired excited state. Now, inversionless amplification of light at the radiative transition to the ground state becomes possible, with the amplified frequency tunable by changing the frequency of the dressing light and selecting an excited state separated from the ground state by the transition frequency. The opening theme of the physics symphony has ascended to a new level.

Clearly, a scheme like that would not work if the dressing light is too weak. But if it is too strong, we may lose excited state population too quickly. It, therefore, makes sense to look at how the whole scheme depends on the interplay of the two fields: the dressing field and the field we are trying to amplify. And thus, a new theme emerges naturally in the symphony, and it takes us to another discovery [24]: there is a balance between the strengths of the two fields when a very stable state is formed, with the lifetime increasing and not decreasing as the intensities of both light fields increase in unison, keeping their ratio constant.

This phenomenon is now recognized in many contexts. For example, it plays important role in autoionization from what should be a dense manifold of rapidly decaying states. The states do not decay, but instead cooperate to create new stable, narrow states at the expense of one that becomes very broad. It has later been re-discovered as interference stabilization of atoms against ionization in strong laser fields, demonstrating its universal character. The same effect is found in waveguides, where the interference of several very leaky modes creates new modes that are stubbornly stable. Mathematical physicists have now developed a sub-field around this genera idea, identifying and exploiting what they call “exceptional points” in the parameter space of non-Hermitian systems. Interference stabilization against ionization in atoms driven by intense light fields also happened to be the topic of my PhD thesis, and at the root of it were AK’s ideas of crafting autoionizing states where one wants them to appear, and the discovery of the unusual, highly counter-intuitive interplay of the ‘leaky’ states.

And thus, the complex and rather abstract idea of exceptional points is traced back to the basic interference of two modes. Through complexity, the original theme of the symphony emerges again, as elegant, as simple, and as beautiful as before.

In science, we always stand on the shoulders of giants. Some are immediately recognized as such, while others accompany us on our science adventures, offering support, encouraging good ideas, helping with deep insight, and with a gentle smile. We may not recognize them for the giants they are and the role they play until they are gone. Then, looking back at the road we travelled together, we understand with striking clarity their lasting impact. Let us take this opportunity to recognize Alexander Popov for the gentle giant he was – as a person, as a physicist, as an artist in his work, and as a tuning fork in ours.

## 4 Nonlinear parametric interactions in gases for generating IR, UV, and VUV

It is unimaginable to write about Alexander Kuz’mich Popov, or Sasha, as I used to call him since we first met 52 years ago, in the past tense. That half-century was filled with true excitement for science and marked by the revolutionary developments and tectonic shifts in Physics, specifically in Optics and Spectroscopy – the field in which Dr. Popov has made foundational contributions. The area of Optics and Spectroscopy deserves the name of the “Queen of Physics” since it enabled early breakthrough discoveries in atomic physics and quantum mechanics. The passionate and inspirational teacher to many of us, Alex Popov will be remembered by the broad optics community for his pioneering contributions to the field of coherent control of atoms and molecules as well as record-breaking achievements in the efficient generation of radiation in resonant gaseous media with wavelengths spanning hard-to-reach spectral ranges such as vacuum ultraviolet (VUV) and far infrared (FIR) [27, 41], [42], [43], [44]. These early pioneering works were performed in Krasnoyarsk, USSR, and were much later recognized by the western community. In year 2000, Dr. Popov moved to the U.S. and extended his research to a totally new and exciting area of optical metamaterials. The exploration of nonlinear optics of metamaterials with a negative index of refraction (negative-index metamaterials - NIMs) opened up new chapters for the science of light (see Section 5 and references therein).

Dr. Popov’s fundamental discoveries and vision have led to the development of practical optical devices including widely used optical parametric oscillators (OPOs) that utilize a nonlinear effect of four-wave mixing in resonant media and can now be found in every optical laboratory [45], [46], [47], [48]. Since the theory of such frequency converters was first developed, it took 40 years till their first experimental demonstration [46], [47], [48] and finally their utilization for the generation of highly entangled photons in resonant Rb vapor, in 2020 [45]. That recent achievement demonstrates how far his scientific vision of future photonic applications has extended.

Alex loved challenges and often tackled exotic, hard-to-achieve projects. He would always work on it vigorously, persistently, and tirelessly. As mentioned above, the results often led to technological success in novel areas of coherent optics, even though the recognition would come 15–20 years later and, in some cases, accompanied by a “reinvention” by other groups.

When we first met, I completed my Master’s thesis, and Alex finished his three-year postdoctoral research in the newly established research group of S.G. Rautian in Novosibirsk, Institute of Semiconductor Physics. The group was studying sub-Doppler width resonances resulting from the interaction of the coherent bi-harmonic fields with an ensemble of free-moving atoms in low-pressure gases. Most of the experiments in that field were limited to the spectroscopy of different transitions in Ne atoms with the aid of the “pump” He–Ne laser and small single-frequency “probe” laser. Very little was known back then on the natural width of the individual atoms and exited states’ decay time and their dependence on collisional processes between Ne atoms. Building on his Ph.D. thesis on the quasi-classical theory of gas lasers Alex in collaboration with S.G. Rautian’s team predicted nonlinear interference structures in the emission and absorption spectra that result from a different state entangled by the external coherent laser fields. This seminal work led to many discoveries and advances including the development of Doppler-free two-photon spectroscopy and new precise measurement of the Lamb shift in Hydrogen atoms, amplification without inversion due to the negative absorption at a range of the emission frequencies, “slow light”, supersensitive magnetometry, and “dark” states.

Interestingly, similar research was done by W.E. Lamb, M. Feld, and P. Toscheck with his graduate student T. Hansch. Except for the fundamental paper of W. Lamb on the theory of “mode pulling” in gas lasers very little was known or studied about cubic nonlinear susceptibility of gases, albeit the computations with the density matrix kinetic equations were straightforward and well described in the literature.

It is worth noting that the only commercially available laser in the Soviet Union in 1970 was a 10 mW He–Ne laser. Only few places in the country built in-house ruby and Nd-YAG lasers, and the only tunable laser radiation was available from sophisticated optical parametric oscillators, which utilized quadratic nonlinearity of optical crystals.

Being Dr. Popov’s first graduate student and collaborator, I was privileged to be taught by Sasha. He most enthusiastically taught his students about everything he knew about atomic spectroscopy, specifically on interesting and emerging topics of atomic transitions, collisional and Doppler broadening that he himself learned from his great teachers A.P. Kazantsev and S.G. Rautian. One characteristic example about Alex’s teaching was when I showed him my M.S. Thesis on “Two- and three-level system’s dynamics in the external fields in the pseudo-Hermitian Quantum Mechanics”. It stemmed from my studies of the Lie model in quantum field theory and was a naive attempt to describe the decay of atomic discrete states with the evolution operator. I proudly showed him my mostly mathematical paper. Even though Sasha was not necessarily interested in pure mathematical derivations, within one week from our first brief discussion, he came up with a detailed written assignment. It contained references to the original Lamb’s paper on phase fluctuations and combination tones oscillations in gas laser, and he explained to me in simple words how to develop the perturbation theory to the third order of nonlinearity. He also presented me with N. Bloembergen’s classic book “Nonlinear Optics”, which became my daily reading for the next few years.

Alex’s life is over 50 years of exciting discoveries in the field of nonlinear spectroscopy. Every work was foundational and addressed a new and exciting effect: level manifolds with different magnetic sublevels, Hanle type Doppler-free spectroscopy in external magnetic field, collision-induced four-wave mixing. The latter is a pioneering study conducted in 1978 that was later experimentally investigated by Popov’s team, when direct measurement a collisional cross-section was made possible by a heterodyning of an extremely weak signal (of the order of 10^−15^ W) generated in the process of the four-wave mixing process. His studies of the efficient VUV generation in gaseous media, enhancement of third harmonic generation with coherent field induced autoionization and observation of collision-induced nonlinear processes was followed by many groups.

Alex’s exploration of remarkable nonlinear effects in NIMs and other composite materials is yet to lead to technology demonstrations that could come to life with the emerging advances in material science and novel methods to overcome intrinsic high losses, in other words, material’s light absorption. Up to now, established and emerging technologies such as the creation of entangled photons for quantum communications in degenerate 4-wave mixing process [45] and frequency comb generation in nonlinear fibers for high-capacity fiber communication channels, built upon Dr. Popov’s seminal results. I believe that the realization of novel optical devices would for long be fed from Popov’s vision of the future of nonlinear optics.

## 5 Theory of nonlinear optics in negative-index metamaterials

Alex Popov made pioneering contributions to the theory of nonlinear optical effects in a special class of artificially engineered optical media, namely, negative-index metamaterials. The refractive index defines the ratio by which the phase velocity of light slows down in a material compared to that in vacuum and normally it is positive in naturally occurring materials. In artificially designed and engineered metamaterials, however, one can obtain not only negative permittivity, *ɛ* < 0 (which is typical for example for plasma and metals) but also negative permeability, *μ* < 0, resulting in a negative refractive index, $n=\sqrt{ɛ}\mu <0$ . Negative-index metamaterials (NIMs) (see for example [49]) can enable a number of counter-intuitive phenomena, such as for example highly discussed and debated super-resolution effect [50] and new guiding and light controlling systems [49]. Interestingly and counter-intuitively, in NIMs, the light phase and the flux of energy defined by the Poynting vector ***S*** are propagating in opposite directions. This is because *n* < 0 results in the direction change of the wavevector **k**, which is proportional to the refractive index *n*: *k* = *nω*/*c*, and **k** defines the phase propagation.

Even in the early years of the field of metamaterials, when various groups around the globe were racing for the first experimental demonstrations of negative-index metamaterials, it became clear that nonlinear optical properties of NIMs should be drastically different from conventional homogenous and composite materials. Let’s consider nonlinear parametric processes, such as second-harmonic generation (SHG) where in one elementary act two photons at frequency *ω* are annihilated and one photon at 2*ω* is created so that the photon energy is conserved. In addition to the photon energy conservation, the photon momentum has to be also conserved so that 2**k**(*ω*) = **k**(2*ω*) (this is also referred to as the phase-matching condition). Normally, when all refractive indexes are positive the fundamental beam at frequency *ω* and the SHG wave at 2*ω* both propagate in the same direction. In NIMs however one can have a situation when *n*(*ω*) < 0, whereas *n*(2*ω*) > 0. In this case, we still need the condition 2**k**(*ω*) = **k**(2*ω*) required by the phase matching to be fulfilled so that the wavevectors for both the fundamental beam and for the SHG wave are in the same direction, whereas the energy for the fundamental beam and for the SHG wave will propagate in the opposite directions. This is because *n*(*ω*) < 0 causes **S**(*ω*) to be anti-parallel to **k**(*ω*), whereas for SHG, **S**(2*ω*) is still in the same direction as **k**(2*ω*). Alex Popov (together with one of the authors, VMS) derived the important Manley-Rowe relations for NIMs, describing how the exchange of energies between the interacting waves evolves in various parametric processes [37]. For example, for SHG, where the generated 2*ω* propagates against the fundamental beam *ω*, i.e. in the reflected direction, it turns out that the difference in the field amplitude squared for the two waves remains the same along the nonlinear medium [37], which is in stark contrast to the conventional positive-index materials, where the sum of these quantities remain constant in lossless media. Other seminal early studies on nonlinear interactions in NIMs can be found in [51], [52], [53]. Dr. Popov with his collaborators also suggested how losses can be compensated in NIMs by optical parametric amplification [36] and discovered several unique features for nonlinear optical phenomena in NIMs (see for example [35, 54], [55], [56], [57], [58], [59], [60], [61], [62]).

It’s important to note that early Popov’s studies on nonlinear optics in negative index materials later open up new fields for nonlinear optics in metamaterials, such as for example exotic and rather unique nonlinear optical properties in metamaterials with near-zero index (NIZ) also referred to as epsilon near-zero (ENZ) materials (see recent review [63]).

## 6 Optical properties of fractal aggregates of colloidal particles

In the late 1980s and early 1990s, together with his collaborators, Alex Popov made seminal contributions to the optics of fractal aggregates of colloidal particles. Random composites, such as aggregates of colloidal metal particles often have the scale-invariant, fractal morphology that enables truly unique optical properties. Specifically, the fractal morphology promotes localization of collective plasmon excitations in small nm-scale areas, referred to as “hot spots”, where the local fields can significantly exceed the field intensity of incident light enabling dramatically enhanced nonlinear optical responses [34]. Importantly, the resonant frequencies of these localized plasmon modes depend on the local geometry and arrangement of particles in various small areas of the fractal, and their excitation depends on the light polarization. As a result, light at different wavelengths and polarizations would excite different distributions of the hot spots, representing the plasmon modes localized in the areas resonating at the given wavelength and polarization of light. If the enhanced field intensities in the hot spots exceed a certain threshold then local photo-modification can occur (typically, due to the sintering process), which is wavelength- and polarization-selective [64]. This enables spectrally and polarization sensitive recording information in deeply sub-wavelength nm-scale areas [64].

Since these early pioneering experimental studies of optical properties of fractal aggregates of colloidal particles by Prof. Popov and his colleagues, the field of random composite materials, such as fractal composites and percolation films, became a hot field where many novel phenomena and applications have been demonstrated (see [65]). This includes for example surface-enhanced Raman scattering (SERS) in fractal structures [66] and, most recently, laser color printing in semi-continuous metal films [67]. Yet again, pioneering and visionary studies conducted by Alex Popov and his colleagues were well ahead of their time and have led to new discoveries for many years.

## 7 Light-induced drift

The phenomenon of light-induced drift (LID) in gases was discovered by Gel’mukhanov and Shalagin in 1979 [68, 69]. Alex Popov with his colleagues also made early important contributions to this unique phenomenon [70, 71]. The essence of LID consists in the creation of directed macroscopic motion of gas during its interaction with a traveling electromagnetic wave, which is at a quasi-resonance with a certain atomic (or molecular) transition between the ground and excited upper state. Here the presence in the system of an extraneous (buffer) gas that does not couple to light is essential.

The appearance of the drift is due to the selective – with respect to the velocities – optical excitation through the Doppler effect of the absorbing-gas atoms and the fact that the atoms in the excited state and those in the ground state elastically collide with the buffer-gas particles at different rates. Under optimal conditions, the LID velocity constitutes an appreciable fraction of the characteristic thermal velocities **v**_th_ so that typical acquired momentum m**v**_th_ in LID is much larger than the photon momentum *ℏ* **k**. Thus, the LID can be much stronger than the radiation pressure. The direction of the drift coincides with, or is opposite to, the direction of propagation the radiation, depending on the ratio of the rates of collisions in the upper and lower states and the sign of the detuning of the radiation frequency from the frequency of the corresponding atomic transition.

LID is enabled by the selection of atoms with particular velocities (through the Doppler effect) and in that regard it is similar to the famous “Maxwell’s Demon”. In fact, one could consider this interaction as the one caused by the entropy exchange between light and gas. Specifically, light which is initially propagating in a particular direction gets then scattered by atoms in all the directions so that light’s entropy increases in the process of LID. In contrast, the gas which is originally completely random and then the directed flux of the absorbing gas is created so that the entropy of gas system decreases, at the expense of the increased entropy of light.

Originally, the LID effect was proposed for the case when the inhomogeneous Doppler broadening significantly exceeds the homogeneous width of the atomic transition so that a relatively small group of atoms is involved into the resonant interaction with light. As Alex Popov and his colleagues showed in [70] one can optimize the effect by using non-monochromatic light with the spectral width comparable to the Doppler width. In this case, a significant fraction of atoms would be involved in the velocity-selective interaction with light.

Alex Popov together with his colleagues also studied LID under the coherent pulsed-periodic excitation, when Rabi oscillations between the two atomic states play an important role. These oscillations are velocity-dependent because the Doppler shift enters into the detuning in the generalized Rabi frequency. Popov and his colleagues showed that in this case the drift direction for LID can be reversed by changing the radiation intensity [71] – the regime which is not possible under the continuous excitation. Professor Popov’s work on the optimization of LID effect under various regimes of excitations, coherent and stochastic, was critically important for further development of various LID related phenomena, such as various light-induced kinetic effects of electrons in solids, which later became the area of active research (see for example [72]).

## 8 The right brain of Alex Popov

### 8.1 On the philosophy of creativity

*Chaadaev, do you remember le temps anciens?*

*A. Pushkin*

The name of Alexander Kuz’mich Popov stands together with the names of the giants of nonlinear optics and spectroscopy – S. A. Akhmanov, N. B. Delone, I. I. Sobelman, V. S. Letokhov, V. P. Chebotaev, S. G. Rautian, and A. M. Shalagin. I (BL) heard his name and knew his reputation already in 1980th, meeting his many students at the famous conferences in Krasnoyarsk and Divnogorsk. But it was only in 2008 that I have met AK personally, at a conference in Porquerolles.

The beautiful island of Porquerolles is situated in Provence at the south of France; one way to reach it is to take a train from Marseille to Hyeres, make your way to the port and take a ferry to the island. A military base there is often used by the French research agencies to host science lectures and workshops. The creative atmosphere and stimulating discussions are helped in no small measure by the good food and even better wine, bringing poetry into the mix of the physics discussions, especially during the dinner, and especially after a glass of red.

Alexander – Sasha – Popov knew poetry as well as physics, and I have to confess that we discussed the former more than the latter. We recited the poetry as an akathist, as a solemn hymn to life around us, as if saying to the breathtaking nature of Provence: “Take us, the holy island dwelling, accept us – the forever homeless wanderers!”

*I was blessed to be born a Russian poet*

*I was honored to be touched by her triumphs*

*But my blessing ran out at birth, Being born in the twenties of the twentieth was my curse*

*Life has blessed – and has cursed me with* everything,

*Like a drunkard, I was cast away*

*On her way from a triumph to victory, I was left in a ditch on a highway*

These verses were written by David Samoilov, and we have recited many of his powerful poems, which shake you from inside out, but then, all of a sudden, the baroque poetry of Igor Severyanin would rise from the emerald Porquerolles’ waves:

*It was all by the sea, in the foam of* *stars*,

*Where the buzz of the city is* *gone*,

*She – the queen – at the piano, playing Chopin’s ballade*

*And her page falling madly in love*

Reciting the poetry brought us together and gave us a chance to connect. With Severyanin’s poetry, Alexander was invariably surprised at how people would utterly denounce it one day and then listen to it in revered silence a couple of years later. He wrote: “I have recently stumbled upon a brilliant recital of Severyanin’s poetry, accompanied by a guitar, a violin, and a piano. I confess – we are nothing but a knot of emotions, and emotions are the same in the 17th and in the 21st century.”

I have recently sent a letter to AK with a quote from the rock-opera “Juno and Avos”1The story of Juno and Avos (“off-chance” in English) is the true story of love between a Russian explorer Nikolai Rezanov and Concepción Argüello, a 15-year-old daughter of the colonial governor of Spanish California. In my mind, this story always connected to the story of love of my friends. composed by Alexey Rybnikov, with the poetry by Andrei Voznesensky:

*We are few, so very few*

*and the worst of it, scattered across the ice*

*of this world, under the Russian cross*

*putting all hopes in a throw of dice*^1^

“Right on!” — replied Alexander, and continued: “By the way, the hero of the Juno and Avos’ story, Nikolai Rezanov, was buried in my home city of Krasnoyarsk, on a peninsula throwing itself into the mighty river of Yenisey. His grave was, of course, washed away. Now there is a concert hall there.” The poetry, continued AK, was written by Andrey Voznesenskiy in a single breath, in a hotel in Los Angeles. Here is its final verse:

*You hoped against all hope*

*To bridge the faraway lands*

*Your hopes and plans were in vain*

*But thanks for the try anyway*.

AK then wrote that, straddling Europe and Asia, Russia was destined to forever be straddled between the two, doomed to geopolitical isolation. Its geography, its climate, and its history of facing such disparate forces as Europe and Genghis Khan’s hordes are at the root of the Russian solitude.

In November 2021 AK turned 80, far from old by today’s standards. The online conference celebrating his birthday brought together many famous scientists from across the world, including his friends from the old days in Krasnoyarsk. The meeting was wonderful, emotional, touching and very informal. In my follow-up letter to AK I wrote that he was a lucky person, who managed to achieve so much and can still achieve a lot more. Quoting Yesenin, a wonderful Russian poet from the turn of the XXth century, I wrote:

*I am a poet, Not a verse-maker, Like others you meet on the way*

*Yes, I may sometimes be drunk*

*But then*

*I can hear the music of stars far away*

And so are you, AK – I continued– you are not like others we meet on the way, you are a poet and not a verse-maker. AK promptly replied: “Salute, Boris, old friend!” and refused to take the compliment by offering a quote from “The Idiot” by Dostoevsky: “One evening, in Basel, in Switzerland, I was shaken awake by the cries of a donkey at the city market. I looked at him and, all of a sudden, everything became crystal clear in my mind.”

AK liked to quote from Pushkin’s “Stories of Ivan Belkin”: “Seriously, what would happen to us if, instead of following the usual rule of respecting the status and the position of a person, we would introduce a different rule – to respect a person’s mind? How many disastrous arguments would such rule breed! … and, just to think about it, how would the servants know who should be served the food first?”

Yet, that is exactly the rule that AK and we, scientists, aim to follow, and it does not lead to problems described by the hero of the “Stories of Ivan Belkin.” One might argue that the reason could be that the left and right halves of a brain are different and we, physicists, use the different half of the brain than writers. However, Alexander Popov used both parts of his brain in equal measure. His creativity was born at the interface of poetry and physics, taking strength in science as much as in art.

My sorrow is light, as writing these notes I speak to Alexander Popov once again, and I hear his voice in response. I have not made it to your Florida home, and we did not have a chance to go together to the islands favored by Hemingway, but in my mind we are already on the way there …

## References

[1] Popova T. Y., Popov A. K., Rautian S. G., Sokolovskii R. I. (1969). Nonlinear interference effects in emission, absorption, and generation spectra. J. Exp. Theor. Phys..

[2] Arkhipkin V., Heller Y. (1983). Radiation amplification without population inversion at transitions to autoionizing states. Phys. Lett. A.

[3] Harris S. E. (1989). Lasers without inversion: interference of lifetime-broadened resonances. Phys. Rev. Lett..

[4] Scully M. O., Zhu S. Y., Gavrielides A. (1989). Degenerate quantum-beat laser: lasing without inversion and inversion without lasing. Phys. Rev. Lett..

[5] Zhu S. Y., Scully M. O., Fearn H., Narducci L. M. (1992). Lasing without inversion. Z. Phys. D.

[6] Alzetta G., Gozzini A., Moi L., Orriols G. (1976). An experimental method for the observation of r.f. transitions and laser beat resonances in oriented na vapour. Nuovo Cimento B.

[7] Arimondo E., Orriols G. (1976). Nonabsorbing atomic coherences by coherent two-photon transitions in a three-level optical pumping. Lett. Nuovo Cimento.

[8] Kocharovskaya O. A., Khanin Y. I. (1988). Coherent amplification of an ultrashort pulse in a three-level medium without a population inversion. JETP Lett..

[9] Kocharovskaya O., Mandel P. (1990). Amplification without inversion: the double- Λ scheme. Phys. Rev. A.

[10] Boller K. J., Imamoğlu A., Harris S. E. (1991). Observation of electromagnetically induced transparency. Phys. Rev. Lett..

[11] Harris S. E. (1997). Electromagnetically induced transparency. Phys. Today.

[12] Hau L. V., Harris S. E., Dutton Z., Behroozi C. H. (1999). Light speed reduction to 17 meters per second in an ultracold atomic gas. Nature.

[13] Rostovtsev Y., Kocharovskaya O., Welch G. R., Scully M. O. (2002). Slow, ultraslow, stored, and frozen light. Opt. Photonics News.

[14] Fleischhauer M., Lukin M. D. (2000). Dark-state polaritons in electromagnetically induced transparency. Phys. Rev. Lett..

[15] Fleischhauer M., Lukin M. D. (2002). Quantum memory for photons: dark-state polaritons. Phys. Rev. A.

[16] Harris S. E., Sokolov A. V. (1998). Subfemtosecond pulse generation by molecular modulation. Phys. Rev. Lett..

[17] Sokolov A. V., Shverdin M. Y., Walker D. R. (2005). Generation and control of femtosecond pulses by molecular modulation. J. Mod. Opt..

[18] Voronine D. V., Sinyukov A. M., Hua X. (2012). Time-resolved surface-enhanced coherent sensing of nanoscale molecular complexes. Sci. Rep..

[19] Scully M. O., Kattawar G. W., Lucht R. P. (2002). FAST CARS: engineering a laser spectroscopic technique for rapid identification of bacterial spores. Proc. Natl. Acad. Sci. U. S. A..

[20] Pestov D., Murawski R. K., Ariunbold G. O. (2007). Optimizing the laser-pulse configuration for coherent Raman spectroscopy. Science.

[21] Scully M. O., Zubairy M. S., Agarwal G. S., Walther H. (2003). Extracting work from a single heat bath via vanishing quantum coherence. Science.

[22] Popova T. Y., Popov A. K. (1970). Effect of resonance radiative processes on the amplification factor. J. Appl. Spectrosc..

[23] Popova T. Y., Popov A. K. (1970). Shape of the amplification line corresponding to an adjacent transition in a strong field. Sov. Phys. J..

[24] Heller Y., Popov A. (1976). Laser-induced narrowing of autoionizing resonances studied by the method of parametric generation. Phys. Lett. A.

[25] Heller Y., Popov A. (1976). Parametric generation and absorption of tunable vacuum-ultraviolet radiation controlled by laser-induced autoionizing-like resonances in the continuum. Opt. Commun..

[26] Popov A. K. (1983). Introduction in Nonlinear Spectroscopy.

[27] Arkhipkin V. G., Popov A. K. (1987). Nonlinear optics and transformation of light in gases. Sov. phys., Usp..

[28] Popov A. K. (1996). Amplification without inversion and laser induced transparency on discrete transition and transition in continuum. Izv. Akad. Nauk, Ser. Fiz..

[29] Popov A. K., Slabko V. V. (2005). Inversionless amplification by anisotropic molecules. Opt. Lett..

[30] George T. F., Popov A. K. (2000). Coherence-controlled transparency and far-from-degenerate parametric gain in a strongly absorbing Doppler-broadened medium. Opt. Lett..

[31] Popov A. K., Myslivets S. A., George T. F. (2005). Nonlinear interference effects and all-optical switching in optically dense inhomogeneously broadened media. Phys. Rev. A.

[32] Heller Y., Popov A. (1976). Autoionizing-like resonances induced by a laser field. Opt. Commun..

[33] Dimov S. S., Pavlov L. I., Stamenov K. V., Heller Y. I., Popov A. K. (1983). Laser-induced nonlinear resonances in the continuum at third-harmonic generation in na vapor. Appl. Phys. B.

[34] Butenko A. V., Chubakov P. A., Danilova Y. E. (1990). Nonlinear optics of metal fractal clusters. Z. Phys. D.

[35] Popov A. K. (2010). Nonlinear optics of backward waves and extraordinary features of plasmonic nonlinear-optical microdevices. Eur. Phys. J. D.

[36] Popov A. K., Shalaev V. M. (2006). Compensating losses in negative-index metamaterials by optical parametric amplification. Opt. Lett..

[37] Popov A. K., Shalaev V. M. (2006). Negative-index metamaterials: second-harmonic generation, Manley Rowe relations and parametric amplification. Appl. Phys. B.

[38] Khanin Y. I., Kocharovskaya O. A. (1990). Inversionless amplification of ultrashort pulses and coherent population trapping in a three-level medium. J. Opt. Soc. Am. B.

[39] Richter M., Lytova M., Morales F. (2020). Rotational quantum beat lasing without inversion. Optica.

[40] Heller Y., Lukinykh V., Popov A., Slabko V. (1981). Experimental evidence for a laser-induced autoionizing-like resonance in the continuum. Phys. Lett. A.

[41] Barantsov V. I., Popov A. K., Tartakovskii G. K. (1975). Possibility of four-photon parametric generation of light in gases. J. Exp. Theor. Phys..

[42] Popov A. K., Timofeev V. P. (1977). Up-conversion with resonantly two-photon pumped atomic nonlinear media. Opt. Commun..

[43] Slabko V. V., Popov A. K., Lukinykh V. F. (1978). Generation of coherent radiation at 89.6 nm through two-photon resonant phase-matched tripling of fourth-harmonic Nd: glass laser radiation in hg vapors. Appl. Phys..

[44] Popov A. K. (1979). Conversion of ir radiation by methods of nonlinear resonance optics. Izv. AN SSSR, Ser. Fiz..

[45] Guerrero A. M., Nussenzveig P., Martinelli M., Marino A. M., Florez H. M. (2020). Quantum noise correlations of an optical parametric oscillator based on a nondegenerate four wave mixing process in hot alkali atoms. Phys. Rev. Lett..

[46] Okuma J., Hayashi N., Fujisawa A., Mitsunaga M., Harada K. I. (2009). Parametric oscillation in sodium vapor by using an external cavity. Opt. Lett..

[47] Yu X., Xiao M., Zhang J. (2010). Triply-resonant optical parametric oscillator by four-wave mixing with rubidium vapor inside an optical cavity. Appl. Phys. Lett..

[48] Sheng J., Wu H., Yang X., Khadka U., Xiao M. (2012). Noise correlations in a doubly-resonant atomic optical parametric oscillator. Opt. Lett..

[49] Shalaev V. M. (2007). Optical negative-index metamaterials. Nat. Photonics.

[50] Pendry J. B. (2000). Negative refraction makes a perfect lens. Phys. Rev. Lett..

[51] Zharov A. A., Shadrivov I. V., Kivshar Y. S. (2003). Nonlinear properties of left-handed metamaterials. Phys. Rev. Lett..

[52] Lapine M., Gorkunov M. (2004). Three-wave coupling of microwaves in metamaterial with nonlinear resonant conductive elements. Phys. Rev. E.

[53] Agranovich V. M., Shen Y. R., Baughman R. H., Zakhidov A. A. (2004). Linear and nonlinear wave propagation in negative refraction metamaterials. Phys. Rev. B.

[54] Popov A. K., Myslivets S. A., George T. F., Shalaev V. M. (2007). Four-wave mixing, quantum control, and compensating losses in doped negative-index photonic metamaterials. Opt. Lett..

[55] Popov A. K., Myslivets S. A. (2008). Transformable broad-band transparency and amplification in negative-index films. Appl. Phys. Lett..

[56] Popov A., Myslivets S., Shalaev V. (2009). Resonant nonlinear optics of backward waves in negative-index metamaterials. Appl. Phys. B.

[57] Popov A. K., Myslivets S. A., Shalaev V. M. (2009). Coherent nonlinear optics and quantum control in negative-index metamaterials. J. Opt. A.

[58] Popov A. K., Myslivets S. A., Shalaev V. M. (2009). Microscopic mirrorless negative-index optical parametric oscillator. Opt. Lett..

[59] Popov A. K., Myslivets S. A., Shalaev V. M. (2010). Coherent nonlinear-optical energy transfer and backward-wave optical parametric generation in negative-index metamaterials. Phys. B: Condens. Matter.

[60] Shalaev M. I., Myslivets S. A., Slabko V. V., Popov A. K. (2011). Negative group velocity and three-wave mixing in dielectric crystals. Opt. Lett..

[61] Popov A. K., Myslivets S. A. (2011). Nonlinear-optical metamirror. Appl. Phys. A.

[62] Popov A. K., Nefedov I. S., Myslivets S. A. (2017). Hyperbolic carbon nanoforest for phase matching of ordinary and backward electromagnetic waves: second harmonic generation. ACS Photonics.

[63] Fruhling C., Ozlu M. G., Saha S., Boltasseva A., Shalaev V. M. (2022). Understanding all-optical switching at the epsilon-near-zero point: a tutorial review. Appl. Phys. B.

[64] Karpov A., Popov A., Rautian S. (1988). Observation of a wavelength- and polarization-selective photomodification of silver clusters. JETP Lett..

[65] Shalaev V. M. (2000). Nonlinear optics of random media: fractal composites and metal-dielectric films. Springer Tracts in Modern Physics.

[66] Stockman M. I., Shalaev V. M., Moskovits M., Botet R., George T. F. (1992). Enhanced Raman scattering by fractal clusters: scale-invariant theory. Phys. Rev. B.

[67] Chowdhury S. N., Nyga P., Kudyshev Z. A. (2021). Lithography-free plasmonic color printing with femtosecond laser on semicontinuous silver films. ACS Photonics.

[68] Gel’mukhanov F. K., Shalagin A. M. (1979). Light-induced diffusion of gases. JETP Lett..

[69] Antsygin V. D., Atutov S. N., Gel’mukhanov F. K., Telegin G. G., Shalagin A. M. (1979). Light-induced diffusion of sodium vapor. JETP Lett..

[70] Popov A. K., Shalagin A. M., Shalaev V. M., Yakhnin V. Z. (1981). Gas drift induced by nonmonochromatic light. J. Exp. Theor. Phys..

[71] Popov A. K., Shalaev V. M., Yakhnin V. Z. (1982). Light-induced gas drift under conditions of pulsed periodic excitation. J. Exp. Theor. Phys..

[72] Shalaev V. M., Douketis C., Stuckless J. T., Moskovits M. (1996). Light-induced kinetic effects in solids. Phys. Rev. B.

